# Caloric-vHIT Dissociation in Vestibular Migraine: An Observational, Retrospective Case Series Study

**DOI:** 10.3390/jcm15145369

**Published:** 2026-07-09

**Authors:** Carlos Palomino-Diaz, Melissa Blanco-Pareja, Margarita Sánchez-Del-Río, Raquel Manrique-Huarte, Pablo Irimia-Sieira, Nicolás Pérez-Fernández

**Affiliations:** 1 Department of Otorhinolaryngology, Clínica Universidad de Navarra, Calle Marquesado de Santa Marta, 1, 28027 Madrid, Spain; cpalominodi@external.unav.es (C.P.-D.); mblancop@unav.es (M.B.-P.); 2 Department of Neurology, Clínica Universidad de Navarra, Calle Marquesado de Santa Marta, 1, 28027 Madrid, Spain; msanchezd@unav.es; 3 Department of Otorhinolaryngology, Clínica Universidad de Navarra, Av. Pío XII 36, 31008 Pamplona, Spain; rrmanrique@unav.es; 4 Department of Neurology, Clínica Universidad de Navarra, Av. Pío XII 36, 31008 Pamplona, Spain; pirimia@unav.es

**Keywords:** vestibular migraine, Ménière’s disease, caloric test, video head impulse test, caloric-vHIT dissociation

## Abstract

**Background**: Caloric–vHIT (CalHiT) dissociation has been considered highly characteristic of Ménière’s disease (MD), but its occurrence in vestibular migraine (VM) remains incompletely characterized. **Objective**: The primary aim was to characterize, through detailed longitudinal analysis, the clinical and mechanistic heterogeneity underlying caloric-vHIT dissociation in seven patients with definite VM in a retrospective cohort. **Study design**: Observational, retrospective case series. **Methods**: From a standardized vestibular cohort of 100 consecutive patients with definite MD or VM evaluated between 2015 and 2018, seven patients with definite VM who exhibited caloric-vHIT dissociation were identified and followed up for approximately seven years. The frequency of dissociation patterns within the source cohort was recorded to contextualize case selection, and each case was characterized descriptively as follows: Patterns were classified as CalHiT-0 (CP < 22%, vHIT ≥ 0.80), CalHiT-A (CP ≥ 22%, vHIT ≥ 0.80), CalHiT-B (CP < 22%, vHIT < 0.80), and CalHiT-C (CP ≥ 22%, vHIT < 0.80). **Results**: At the cohort level, A-pattern dissociation occurred in 17.5% of patients with VM (7/40) and 56.7% of patients with MD (34/60), while B-pattern dissociation was rare (MD: 2/60, 3.3%; VM: 1/40, 2.5%). One patient with CalHiT-A VM was lost to follow-up and excluded from the longitudinal analysis. At the case level, the seven cases (six CalHiT-A and one CalHiT-B) suggested mechanistic heterogeneity, including compensated peripheral hypofunction, familial vulnerability, traumatic injury, and potential central modulation. VM was distinguished by the absence of fluctuating auditory symptoms, absence of progressive hearing loss characteristic of MD, and DHI-measured disability comparable to that of MD despite substantially lower objective vestibular deficits. No VM case with confirmed follow-up evolved toward MD. **Conclusions**: Caloric-vHIT dissociation occurred in approximately one-sixth of patients with definite VM and should not be regarded as a stand-alone sign of endolymphatic hydrops. The clinical heterogeneity across cases suggests that several mechanisms other than hydrops may produce a comparable pattern. These observations could support future studies exploring whether a distinct VM phenotype defined by this dissociation can be established and favor interpreting the dissociation alongside the auditory and longitudinal profiles rather than in isolation.

## 1. Introduction

Vestibular migraine (VM) has been extensively studied in recent years, particularly because of its prominent vestibular symptoms, which can be mistaken for other episodic vertigo disorders that pose diagnostic challenges. It has a high lifetime prevalence, affecting approximately 7% of patients with vertigo and 14% of those with migraine [1]. VM is among the most common causes of recurrent vertigo and exerts a substantial impact on patients’ quality of life [2]. Nevertheless, only approximately one-third of individuals fulfill the established clinical criteria for VM [3].

A female predominance is consistently described, with sex ratios reported between roughly 1.5:1 and 5:1 [1]. VM accounts for 12.5% of diagnoses in specialized clinics, and follow-up studies indicate that up to 37% of cases occur in postmenopausal women [4,5]. Migraine onset typically precedes vestibular symptoms by a mean of nine years [6]. Long-term follow-up extending up to nine years has demonstrated persistent vertigo with decreasing frequency and severity over time, although complete resolution occurs in only 5.4% of patients [7,8].

Beyond vestibular symptoms, auditory manifestations can emerge or intensify over time; tinnitus and aural fullness occur in up to 49% and 50% of VM patients, respectively, while mild bilateral low-frequency sensorineural hearing loss affects 12–18% of cases, with more than half subsequently developing MD during extended follow-up [7,8]. Aural fullness and ictal tinnitus, hallmark features of MD, occur with a notable frequency in VM (exceeding 40%) compared with MD (approximately 80%). Subjective ictal sensorineural hearing loss occurs in 37% of VM patients versus 80% MD patients [9]. Unlike MD, sensorineural hearing loss in VM manifests as mild, bilateral, low-frequency impairment that rarely progresses to profound levels and generally remains transient and reversible [9,10]. This heterogeneity has prompted nosological designations such as “cochlear migraine phenotype” and “cochleovestibular migraine” [11,12].

Quantitative vestibular testing contributes meaningfully to separating the two entities. Canal paresis (CP) occurs in 10–20% of VM adults versus 48–67% of MD [13,14]. Simultaneous directional preponderance and canal paresis occur in approximately 6% of VM versus 15% of MD cases [15]. Caloric testing applies a non-physiological, low-frequency thermal stimulus and is relatively time-consuming, yet it remains highly sensitive for detecting milder vestibular asymmetries [16]. VM is characterized by enhanced sensitivity to vestibular stimulation [17], and patients experience more pronounced autonomic symptoms during caloric testing than those with other vestibular disorders [18]. In addition, caloric stimulation itself may precipitate migraine attacks in susceptible individuals, suggesting that responses to caloric testing reflect disease-specific sensory processing in VM [18].

The video head impulse test (vHIT) demonstrates valuable diagnostic differentiation with a positive predictive value of 81%; abnormal horizontal findings occur in approximately 7% of patients with VM versus 29% of patients with MD, with corrective catch-up saccades in 12% versus 29% of patients, respectively [9]. Notably, the vHIT results typically remain normal during interictal periods [19]. MD depends substantially on disease stage; with progression, caloric deficits may also be observed during interictal periods [20]. Although caloric hypofunction typically favors MD, the etiology of caloric abnormalities in VM remains a subject of debate because the symptoms of MD and VM substantially overlap, and differentiation remains challenging in some cases, underscoring the need to enhance diagnostic capabilities. The proposed mechanisms include caloric hyper-responsiveness arising from relative vestibular asymmetries [19] or potentially central modulation [21].

Caloric-vHIT dissociation, characterized by abnormal caloric responses with a normal vHIT, is considered a hallmark of MD, whereas abnormal vHIT findings suggest peripheral vestibular lesions. However, this pattern is not exclusive to the MD. The primary objective of this study was to characterize, through a detailed longitudinal case series, the clinical phenotype, vestibular and audiological findings, and long-term course of patients with definite VM who exhibited caloric-vHIT dissociation.

## 2. Materials and Methods

### 2.1. Study Design and Rationale

This was an observational retrospective case series. From a standardized vestibular database of 100 consecutive patients with definite MD or VM evaluated between 2015 and 2018, seven patients with definite VM who presented caloric-vHIT dissociation and had complete longitudinal data (approximately seven years of follow-up) were identified and constituted the case series. The frequency of caloric-vHIT dissociation patterns within the source cohort was recorded solely to contextualize case selection and to indicate how often this pattern occurred in each diagnostic group. This cohort-level information was reported descriptively and was not used for between-group comparisons. Case-level observations are offered as descriptive and hypothesis-generating material to guide subsequent studies.

The study was conducted jointly by the Departments of Otorhinolaryngology and Neurology at Clínica Universidad de Navarra (Spain). Ethical approval was granted by the institution’s Research Ethics Committee of the University of Navarra (CEI-UN; Approval Code 70/2016, 7 June 2016; and protocol 2025.308, 11 December 2025), and all procedures adhered to the principles of the Declaration of Helsinki. Given the retrospective design of the study, the requirement for informed consent was waived. This cohort has been described previously in a companion publication [22] that addressed a distinct question: patient-level diagnostic modeling of speech discrimination scores and CalHiT-A status through logistic regression. The present study draws on the same baseline vestibular dataset but pursues a fundamentally different aim: the clinical–mechanistic interpretation of caloric-vHIT dissociation in VM through detailed longitudinal case analysis. No inferential modeling was repeated, and no results, figures, or tables were duplicated between the two publications.

### 2.2. Source Population and Selection

The source population comprised all patients evaluated for recurrent vertigo at our center between 2015 and 2018. During this period, every patient underwent the same standardized comprehensive vestibular protocol irrespective of the suspected diagnosis, comprising neurotological examination, vHIT, bithermal caloric testing, ocular and cervical vestibular evoked myogenic potentials (VEMPs), pure-tone audiometry spanning low-to-high frequencies (125 Hz to 8 kHz), and 3-Tesla brain MRI. Patients meeting the Bárány Society/ICHD-3 criteria [23,24] for definite MD [25] or definite VM [23,24] who completed this protocol were eligible; those with incomplete vestibular testing or insufficient follow-up documentation were excluded. The resulting cohort comprised 100 patients (60 with definite MD and 40 with definite VM). The higher proportion of MD relative to VM (60:40) reflects the referral pattern of a tertiary neurotology center.

### 2.3. Diagnostic Stability and Case Selection

**Figure 1:**
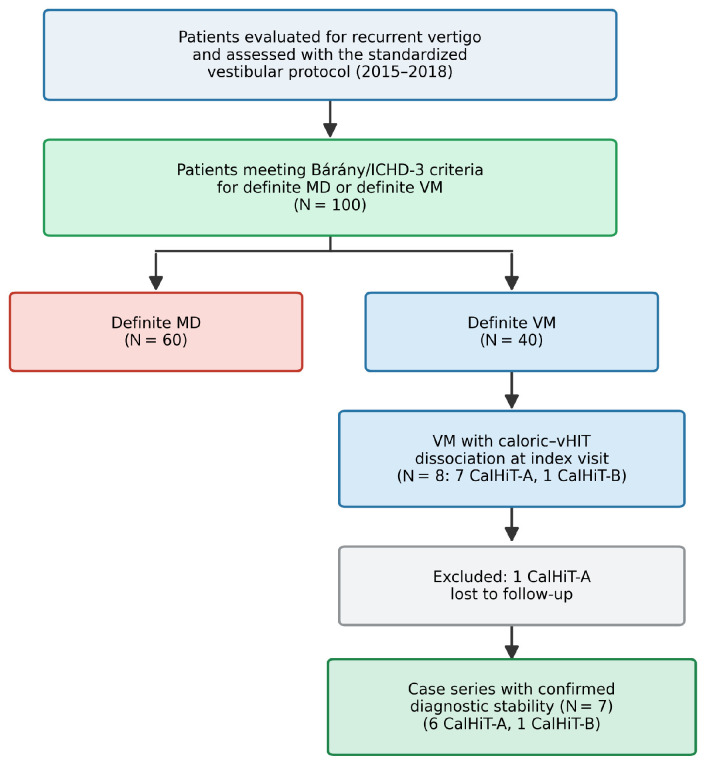
Study flowchart. Derivation of the case series from the source cohort of 100 patients with definite MD or VM evaluated between 2015 and 2018. Of the 40 patients with definite VM, eight exhibited caloric-vHIT dissociation at the index visit (seven CalHiT-A, one CalHiT-B); one CalHiT-A patient was lost to follow-up and excluded, leaving seven cases with confirmed diagnostic stability. MD, Ménière’s disease; VM, vestibular migraine; CalHiT, caloric-vHIT pattern.

Diagnostic stability was confirmed at 6–7 years through in-person clinic visits whenever feasible, and for patients unable to attend, structured interviews were conducted. From the 40 VM patients, eight exhibited caloric-vHIT dissociation at the index visit (seven CalHiT-A and one CalHiT-B). One CalHiT-A patient was lost to follow-up and excluded from the longitudinal analysis (39 evaluable VM patients), leaving seven cases (six CalHiT-A and one CalHiT-B) with confirmed diagnostic stability for the detailed case series (Figure 1). No MD case progressed to bilateral involvement.

### 2.4. Vestibular Testing

Prior to vestibular and audiological testing, all patients underwent a complete otorhinolaryngological examination, including otoscopy and tympanometry, to exclude middle ear pathology. The bithermal caloric test was performed with ICS Chartr 200 (Otometrics, a division of Natus Medical Incorporated, Middleton, WI, USA). Warm (44 °C) and cool (30 °C) water irrigations were delivered to each ear, and the peak response was quantified as the average slow-phase velocity over a 10–s interval of maximal nystagmus. For patients with MD, analyses were conducted separately for the affected and unaffected ears, while for patients with VM, comparisons were made between the right and left ears. The Jongkees formula was used to calculate canal paresis (CP) and directional preponderance (DP) [26]; CP values exceeding 22% and DP values exceeding 28% were classified as abnormal. The video head impulse test (vHIT) was carried out using video-oculography (OTOsuiteV 4.0 Vestibular software: Natus-Otometrics, Taastrup, Denmark) involving brief, sudden, and unpredictable horizontal head impulses (amplitude: 10–20°; peak velocity: >150–200°/s), ensuring a minimum of 20 valid impulses per direction [27]. Normal horizontal canal gain was defined as ≥0.80, and vertical canal gain as ≥0.70. Both caloric testing and the vHIT were performed on the same day at the index visit by a single trained examiner.

### 2.5. Patient-Reported Outcome Measures

Disease-specific handicap and symptom severity were assessed using two validated, self-administered questionnaires, applied at the index visit as part of the standardized vestibular protocol. The Dizziness Handicap Inventory (DHI) is a 25-item patient-reported instrument scored 0–100 across functional, physical, and emotional subscales, with higher scores indicating greater perceived disability from dizziness or vertigo; it is one of the most widely used and validated handicap measures in vestibular disorders. The Vertigo Symptom Scale (VSS) is a self-report questionnaire quantifying the frequency and severity of vertigo-balance and autonomic-anxiety symptoms over a defined recall period, with higher scores reflecting greater symptom burden; the short form (VSS-SF) yields two subscales (vertigo-balance and autonomic-anxiety) that were summed to obtain the severity score reported in this study. Both instruments were administered in their validated Spanish versions.

### 2.6. Caloric-vHIT Dissociation Pattern Classification

The cohort was divided into four groups based on caloric and vHIT outcomes using the CalHiT classification: CalHiT-0 (canal paresis (CP) < 22% with vHIT gain ≥ 0.80), CalHiT-A (CP ≥ 22% with vHIT gain ≥ 0.80), CalHiT-B (CP < 22% with vHIT gain < 0.80), and CalHiT-C (CP ≥ 22% with vHIT gain < 0.80) [22]. Notes: (1) CP was calculated using Jongkees’ formula, with an abnormality cutoff of ≥22%. (2) A horizontal canal vHIT gain below 0.80 was considered abnormal, and vertical canal results were excluded from the CalHiT classification.

### 2.7. Statistical Analysis

The unit of analysis was the individual patient. For the secondary cohort component, we report only descriptive frequencies and proportions of caloric-vHIT (CalHiT) patterns by diagnostic group. Consistent with the contextual role of this component, no hypothesis testing, confidence interval estimation, effect size calculation, or significance threshold was applied in the present analysis. The formal inferential comparison of CalHiT-pattern frequencies between MD and VM, together with the associated diagnostic modeling, is reported in the companion publication [22] and is not repeated here.

## 3. Results

### 3.1. Cohort-Level Frequency of Caloric-vHIT Dissociation Patterns (Contextual)

The study cohort included 60 patients diagnosed with definite unilateral MD and 40 patients with definite VM. Diagnostic stability was confirmed over 6–7 years through in-person clinical evaluations when possible, supplemented by structured telephone interviews for patients who missed recent visits. No cases of MD developed bilateral symptoms, and no VM cases required diagnostic changes after confirmation at follow-up.

Demographic and clinical characteristics of the full cohort have been reported in detail in the companion publication [22]. In brief, VM patients were predominantly female (87.5% vs. 55.0% in MD), the two groups were of similar mean age (MD: 52.3 ± 12.1 years; VM: 48.7 ± 11.8 years), and disease duration was somewhat longer in MD (5.2 ± 6.0 vs. 3.4 ± 3.8 years). Dizziness Handicap Inventory scores (MD: 42.8 ± 18.4; VM: 40.9 ± 27.3), Vertigo Symptom Scale severity scores (MD: 14.2 ± 10.4; VM: 12.4 ± 7.4), and number of crises in the preceding year (MD: 7.2 ± 12.1; VM: 8.8 ± 12.3) were comparable between the groups. Formal between-group comparisons are reported in the companion publication [22] and are not repeated here.

Baseline demographic and clinical characteristics of the two diagnostic groups are summarized in Table 1.

**Figure 2:**
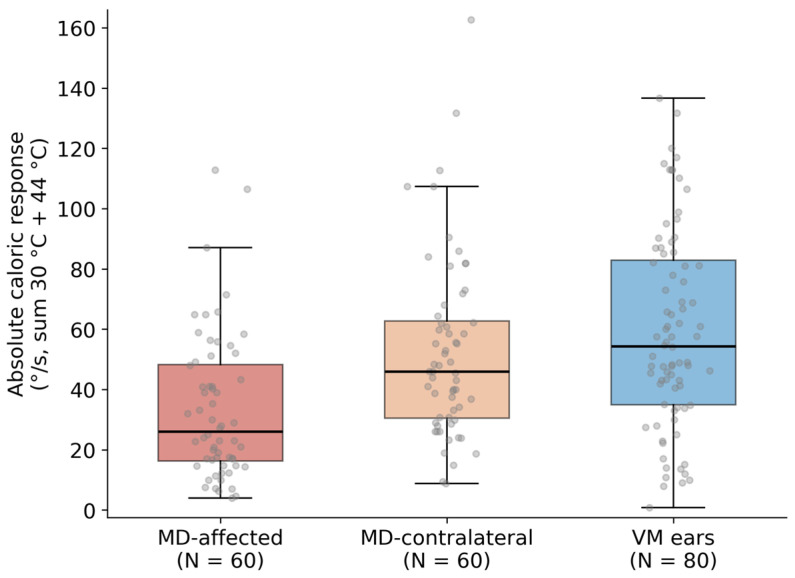
Caloric responses by ear group (mean ± SD) at the index visit (2015–2018). Absolute caloric sum (sum of slow-phase velocities across all four irrigations) is shown for MD-affected ears (N = 60), MD-contralateral ears (N = 60), and VM ears (N = 80; both ears of all 40 VM patients). Individual circles represent single ear-level caloric values overlaid on each box. Descriptive differences are presented in Table 2. The cross-sectional analysis includes the full cohort; longitudinal follow-up was based on 39 evaluable VM patients after exclusion of one patient lost to follow-up. MD, Ménière’s disease; VM, vestibular migraine; SD, standard deviation.

Absolute caloric responses were greater in patients with vestibular migraine (VM) compared to those with Meniere’s disease (MD). At the ear level, ears affected by MD showed a pronounced reduction in caloric response: the mean absolute caloric sum was lowest in MD-affected ears, intermediate in the contralateral ears of MD patients, and highest in ears of VM patients (Figure 2). Descriptive differences are presented in Table 2. In contrast, the vHIT demonstrated smaller overall group differences, with minimal horizontal canal gain asymmetries across both groups, and the majority of VM patients maintained normal vHIT gains (≥0.80) despite variable caloric responses. As shown in Table 2, the mean absolute caloric sum was markedly lower in the MD-affected ears than in the MD-contralateral and VM ears, whereas the MD-contralateral and VM ears differed only modestly.

At the index visit (N = 100), classic A-pattern dissociation (CP ≥ 22% with normal vHIT gain ≥ 0.80) was identified in 34 out of 60 patients with Meniere’s disease (56.7%) and in 7 out of 40 patients with vestibular migraine (17.5%, 7/40). CalHiT-0 (concordant normal) was the predominant pattern in VM (32/40, 80.0%) compared with MD (18/60, 30.0%). Concordant abnormal results (CalHiT-C: CP ≥ 22% with vHIT gain < 0.80) were observed in 6 MD patients (10.0%) and no VM patients. B-pattern dissociation (normal caloric testing with reduced vHIT) was rare in both groups (MD: 2/60 [3.3%]; VM: 1/40 [2.5%]) (Table 3). These findings indicate that classic caloric-vHIT dissociation, although characteristic of MD, can also occur in a clinically relevant subset of patients with VM, creating diagnostic ambiguity.

**Figure 3:**
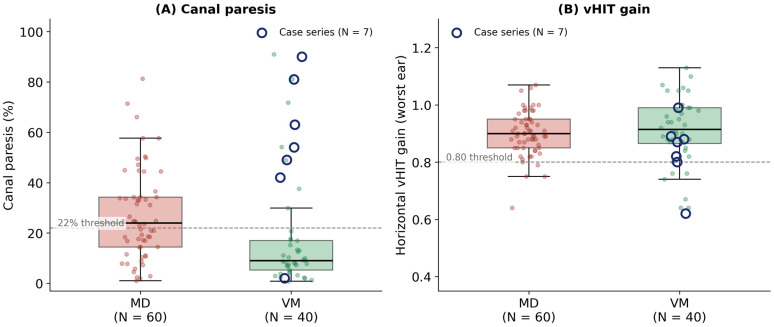
Distribution of canal paresis and vHIT gain by diagnosis. (**A**) Canal paresis (%) and (**B**) horizontal vHIT gain (worst ear) in patients with definite MD (N = 60) and definite VM (N = 40). Within each box, the central line indicates the median and the box limits represent the interquartile range, with whiskers extending to the range excluding outliers. Colored dots represent individual patient values (red, Ménière’s disease; green, vestibular migraine); open navy-blue circles identify the seven vestibular migraine patients comprising the longitudinal case series. Dashed lines mark the abnormality thresholds (canal paresis = 22%; vHIT gain = 0.80). MD, Ménière’s disease; VM, vestibular migraine; vHIT, video head impulse test.

The distributions of canal paresis and horizontal vHIT gain across both groups are shown in Figure 3; no patient exhibited a vHIT gain exceeding 1.2 (Figure 3B).

### 3.2. Case Series: Clinical and Longitudinal Findings (Primary Analysis)

From the 40 VM patients, seven cases (six female, one male; age range 18–69 years) were selected for detailed analysis based on the presence of caloric-vHIT dissociation and complete clinical documentation. The main clinical characteristics and longitudinal phenotypes are summarized in Table 4, and audiovestibular testing is shown in Table 5. Six patients exhibited A-pattern dissociation (CP range: 42–90%), and one exhibited B-pattern dissociation (reduced left horizontal canal vHIT gain of 0.62 with CP 2%).

Case 1 is described as a 69-year-old woman with a long-standing history of low-frequency episodic migraine without aura. In 2016, she experienced the first of several episodes of spontaneous rotational vertigo typically lasting hours, accompanied by intense vegetative symptoms and migrainous features, including headache, photophobia, and phonophobia. After these initial events, the patient remained free of vestibular symptoms for several years, while migraine continued at its baseline frequency. From 2024 onward, she developed monthly recurrent spontaneous vertigo and persistent unsteadiness associated with migrainous features, without fluctuating auditory symptoms; migraine frequency escalated to five episodes per month. At the time, she was receiving preventive treatment with flunarizine and botulinum toxin due to increased migraine recurrence and episodic vertigo. Vestibular testing showed mild bilateral high-frequency sensorineural hearing loss, reduced left horizontal canal vHIT gain (0.62) with normal caloric response (CP, 2%), and normal VEMPs (B-pattern dissociation). Reduced-gain vHIT was accompanied by overt refixation saccades.

Case 2 (65-year-old woman) had migraine without aura (two episodes per month), chronic tension-type headache, a history of recurrent spontaneous vertigo, and disabling imbalance. Her medical history was notable for a traumatic brain injury in 2005, after which she developed positional vertigo. Benign paroxysmal positional vertigo (BPPV) was excluded during positional testing. By 2016, she experienced spontaneous vertigo episodes lasting minutes to hours, which were associated with persistent unsteadiness and falls. Interictally, she reported bilateral tinnitus and aural fullness that did not fluctuate during the attacks. Otoneurological examination revealed spontaneous right-beating nystagmus that was significantly exacerbated following the head-shaking test. During follow-up, vertigo episodes increased in frequency and duration, lasting several hours and leading to further falls without any associated auditory symptoms. Vestibular symptoms were frequently accompanied by migrainous features, including headache, photophobia, and phonophobia, with migraine frequency of 4–5/month. At the time of assessment, the patient had not received any preventive migraine therapy. Vestibular testing revealed canal paresis (CP) of 81%, normal vHIT, and preserved otolithic responses.

Case 3 (a 47-year-old woman) had a strong family history of vestibular disorders (MD in a sibling and VM in her mother). She presented with chronic instability, episodic spontaneous rotational vertigo that typically lasted from minutes to hours, tinnitus (laterality not specified in the available clinical record), and migraine without aura characterized by severe left hemicranial throbbing, lasting 12 to 48 h, with a frequency of four to five episodes per month and catamenial exacerbation. Her clinical course was further complicated by recurrent episodes of BPPV affecting different semicircular canals. These BPPV episodes affected the right posterior and right horizontal canals, occurring as a secondary, interictal condition years after the onset of migraine and vestibular migraine symptoms, rather than preceding them. Despite these symptoms, the patient did not develop fluctuating hearing loss or typical hydropic crisis. Vestibular testing revealed asymmetric caloric responses (CP, 54%), normal vHIT, and a reduced ocular VEMP (oVEMP) on the left side. Recurrent BPPV occurred during interictal periods, with several episodes involving right posterior canal canalithiasis and right horizontal canal cupulolithiasis, and repositioning maneuvers achieved complete resolution of these positional symptoms. Over more than a decade of follow-up, vestibular symptoms decreased in frequency after the introduction of migraine prophylaxis, whereas auditory thresholds remained stable.

Case 4 describes a 50-year-old man with a history of migraine without aura that started and ended during his twenties. He was evaluated in 2015 for episodic spontaneous rotational vertigo accompanied by migrainous features (photophobia and phonophobia) in more than half of the episodes (five to six episodes over six months), as well as positional vertigo. Otoneurological examination revealed spontaneous downbeat nystagmus (DBN), which was significantly exacerbated after the head-shaking test. To date, no fluctuating auditory symptoms have been observed. Vestibular testing revealed isolated unilateral caloric weakness (CP, 42%) and mild high-frequency sensorineural hearing loss in the left ear. The vHIT was normal, with horizontal canal gains of 0.92 (right) and 0.89 (left), consistent with A-pattern dissociation. Vestibular symptoms resolved over time, and at long-term follow-up, the patient remained asymptomatic, without vertigo, imbalance, or migrainous headaches.

Case 5 (an 18-year-old woman) with a history of childhood migraine with visual aura was evaluated in 2015 during pregnancy due to episodic positional dizziness. She reported migraine with and without visual aura occurring two to three times per month, accompanied by episodes of spontaneous vertigo lasting 5 to 10 min and positional vertigo. Otoneurological examination revealed spontaneous DBN and horizontal right-beating nystagmus during head-shaking test. The Dix–Hallpike and supine roll tests were negative, ruling out the presence of BPPV. No auditory symptoms were observed. Vestibular testing revealed unilateral caloric paresis (CP; 63%) and preserved vHIT. At the long-term follow-up, she experienced only sporadic migraine episodes without aura and rare brief spontaneous vertigo, occurring once or twice per year, without recurrence of positional vertigo or auditory complaints.

Case 6 (an 18-year-old woman) with migraine with visual aura was evaluated in 2017 for recurrent spontaneous and positional rotational vertigo that typically lasted 5 to 20 min, and more than half of the episodes coincided with severe migrainous attacks with visual aura. In 2018, the patient was diagnosed with left horizontal canal BPPV and required repositioning maneuvers. This BPPV episode was secondary and interictal, occurring after the established diagnosis of vestibular migraine rather than preceding it. Over time, she developed frequent episodes of instability and spontaneous vertigo several times per week, accompanied by migrainous features without aura or auditory symptoms. Vestibular testing revealed persistent low-frequency canal asymmetry (49%), normal vHIT, and normal otolithic response.

Case 7 (a 52-year-old woman) presented with migraine with visual aura since her thirties. She was referred for evaluation of a five-month progression of daily episodes of head-motion dizziness with nausea lasting hours, accompanied by several migraine features, including phonophobia, photophobia, and visual aura, as well as persistent instability and bilateral tinnitus. Despite marked unilateral caloric paresis (90%), the remainder of the vestibular evaluation was normal. Over the subsequent years, vestibular symptoms remained stable with minimal impact on daily activities, and migraine frequency decreased to approximately one episode per year. She did not present with any cochlear or auditory symptoms other than stable tinnitus.

Cerebral 3T magnetic resonance imaging was unremarkable in all seven cases, with no evidence of posterior fossa abnormalities, white matter lesions, or other structural findings relevant to vestibular symptoms.

Across all seven cases, several common clinical features were observed: absence of progressive or fluctuating sensorineural hearing loss characteristic of MD (mild age-related high-frequency hearing loss was observed in Cases 1 and 4 but did not follow a fluctuating low-to-mid frequency pattern), and symptom severity frequently disproportionate to the measured vestibular deficits. None of the seven VM patients developed definite MD during the observation period (mean follow-up, 7 years).

## 4. Discussion

### 4.1. Clinical Interpretation of Caloric-vHIT Dissociation Patterns in Vestibular Migraine

In summary, caloric-vHIT dissociation was identified in 17.5% of patients with definite VM at the cohort level, occurring at roughly one-third the frequency observed in MD (56.7%), and the seven longitudinally confirmed cases showed marked clinical heterogeneity despite sharing the same A- or B-pattern dissociation, with none progressing to definite MD over a mean follow-up of seven years. These seven cases illustrate the spectrum of caloric-vHIT dissociation encountered in clinical practice and highlight the heterogeneity of the underlying mechanisms. In a large confirmatory cohort (N = 2101), A-pattern dissociation demonstrated high specificity and positive predictive value for distinguishing MD from VM (83.5% and 82.6%, respectively), with a sensitivity of 58.9% in MD and a false-positive rate of 16.5% in VM, although these values were derived using different thresholds (vHIT gain > 0.7 and CP ≥ 25%) [28]. The B-pattern (normal calorics with abnormal vHIT) is exceedingly rare and suggests an alternative peripheral pathology [14]. In our series, caloric-vHIT dissociation was present in all seven patients; six exhibited the classic A-pattern and one demonstrated the B-pattern. Although caloric weakness has conventionally been interpreted as a hallmark of unilateral peripheral vestibular loss, increasing physiological and clinical evidence suggests that this finding, particularly when isolated, may arise from multiple sources and does not necessarily indicate evolving or symptomatic vestibulopathy. A higher prevalence of caloric-vHIT dissociation in MD is expected given its unilateral peripheral nature; however, the finding that approximately one in six VM patients exhibited A-pattern dissociation challenges the assumption that this pattern constitutes de facto evidence of endolymphatic hydrops. A companion analysis of this cohort confirmed that CalHiT-A alone has limited diagnostic power (AUC 0.674) compared with speech discrimination scores (AUC 0.866), supporting its role as an adjudicative rather than a primary diagnostic marker [22]. Our VM prevalence of A-pattern dissociation (17.5%) is consistent with prior series reporting caloric-vHIT dissociation in a minority of VM patients, such as Mavrodiev et al. (false-positive rate 16.5%) [28] and Yilmaz et al. [15], and contrasts with the substantially higher rates reported in MD cohorts.

### 4.2. Frequency-Specific Dissociation Rather than Diagnostic Conflict

The caloric test examines the horizontal canal vestibulo-ocular reflex at ultralow frequencies (<0.01 Hz), whereas the vHIT samples high-frequency function (>5 Hz). Therefore, the dissociation between both tests may reflect frequency-dependent recovery or non-uniform injury across canal afferent populations rather than a diagnostic conflict. Experimental work supports this concept: partial recovery of high-frequency VOR after peripheral loss may normalize the vHIT, whereas caloric responses remain impaired [29]. The disproportionately low caloric output in clinically compensated individuals closely aligns with the patterns observed in several cases.

### 4.3. Stable Compensated Hypofunction Versus Active Disease

- Two phenotypes emerged in this series. Cases 1 and 7 are most consistent with stable compensated unilateral hypofunction. In Case 1, the initial severe vestibular episode may represent a remote unilateral vestibular insult, although this remains speculative because of the lack of contemporaneous documentation. The current vestibular profile does not show classical residual signs of neuritis. However, partial recovery of high-frequency VOR gain and compensatory strategies may obscure prior deficits. The observed findings are compatible with a stable, compensated peripheral lesion, upon which a migraine-related vestibular phenotype acts as the main driver of episodic symptoms. Similarly, in Case 7, despite marked unilateral caloric paresis, the remainder of the vestibular evaluation was normal, and the clinical course was non-progressive, consistent with a remote vestibular insult. The absence of fluctuating auditory symptoms and long-term stability in both cases argue against MD or evolving endolymphatic hydrops. Caloric paresis alone is insufficient to diagnose or infer active inner ear pathology. Beyond the canal paresis value, the slow-phase velocity of each induced caloric response and the directional preponderance should also be considered for a comprehensive interpretation of vestibular function [30].
- Case 2 was related to a structural or traumatic vestibular injury. The temporal relationship between head injury and the vestibular test profile—chronic low-frequency hypofunction with normal vHIT and preserved otolithic responses—supports a diagnosis of post-traumatic labyrinthine concussion, indicating a localized and stable peripheral injury rather than a progressive degenerative or hydropic process. Migraine likely contributes to symptom exacerbation but does not explain the underlying physiological deficit. Case 4 exemplifies the challenge of interpreting isolated caloric results: an isolated unilateral caloric weakness contrasts with the otherwise normal vestibular assessment and the fully compensated clinical course. Technical variability or methodological artifacts cannot be excluded, particularly when caloric abnormalities are not corroborated by deficits in other tests assessing the same end organ. Long-term asymptomatic evolution argues against active inner ear disease [31].

### 4.4. Familial Susceptibility Without Phenotypic Conversion

Case 3 illustrates a clinically relevant phenomenon increasingly recognized in familial vestibular disorders that may appear in first-degree relatives of patients with MD [32], including asymmetric caloric responses and abnormal VEMPs in familial vestibular disorders [33]. This patient has not developed fluctuating auditory symptoms or classical hydropic crises over the past decade, supporting the concept of incomplete penetrance and latent vestibular vulnerability rather than inevitable disease expression. This case may be interpreted as familial vestibular susceptibility, in which subclinical peripheral abnormalities coexist with migraine and recurrent BPPV. Migraine may act as a central amplifier, facilitating symptom expression without causing structural inner-ear disease.

### 4.5. Peripheral Mechanisms: Trigeminal-Vascular Activation and Inner Ear Involvement

Engagement of the trigeminovascular pathway, with consequent liberation of CGRP and related neuropeptides, may impact labyrinthine and retrolabyrinthine structures, such as the spiral ganglion, vestibular nerves, and vestibular nuclei [34]. Two complementary mechanisms have been proposed: CGRP-mediated vasodilation promotes neurogenic inflammation, increased vascular permeability, and fluid extravasation, potentially leading to transient endolymphatic hydrops—supported by MRI evidence in some VM patients [35]; at the same time, serotonergic vasospasm may cause labyrinthine hypoperfusion and cumulative subclinical damage [35]. Furthermore, inflammatory damage to the vestibular nerve may selectively impair the fibers responsible for processing low-frequency stimuli, potentially contributing to the caloric-vHIT dissociation pattern observed in both peripheral vestibulopathy and auditory neuropathy spectrum disorders [36].

### 4.6. Migraine as a Clinical Amplifier of Vestibular Asymmetry

Migraine emerged as a dominant factor in symptom expression across several cases, as illustrated in Cases 5 and 6. In Case 5, the coexistence of transient spontaneous and positional vestibular symptoms, unilateral caloric weakness, and preserved high-frequency VOR responses suggested stable, partially compensated peripheral hypofunction. As a physiological stressor, pregnancy, together with migraine-related central sensitization, may lower the threshold for symptom expression [17]. Studies have indicated that vestibular migraine involves disrupted central processing of signals from the semicircular canals and otolith organs, resulting in altered vestibular integration in affected patients, even in the absence of overt peripheral deficits [21]. This central vestibular hyperexcitability may amplify the clinical expression of subtle peripheral asymmetries that would otherwise remain subclinical [37]. The absence of hearing loss, resolution of positional features, and long-term clinical stability argue against MD. Case 6 illustrates multifactorial vestibular vulnerability, combining persistent low-frequency canal asymmetry, recurrent otoconial instability, and migraine-related central hyperexcitability. Normal vHIT and otolithic responses support a compensated or frequency-limited peripheral substrate, rather than an active degenerative process. The absence of auditory fluctuations and lack of progression toward a Ménière-like phenotype over follow-up are more consistent with a migraine-dominant mechanism, with peripheral asymmetry and recurrent BPPV contributing as facilitators rather than primary drivers [38]. These mechanisms can unmask or exaggerate the symptoms arising from mild or compensated peripheral deficits. These observations may reflect an interaction between migraine-related central processing and subclinical peripheral asymmetries, although this remains hypothetical.

### 4.7. The Role of Velocity Storage in Caloric-vHIT Dissociation

The velocity storage integrator (VSI), comprising the vestibular nuclei, nodulus, uvula, and nucleus prepositus hypoglossi, and receiving modulatory input as well as excitatory projections from migraine-related brainstem regions [39], may play a key role in this dissociation. Caloric testing stimulates primary vestibular afferent neurons with regular and irregular resting discharge patterns. During migraine episodes, excitatory brainstem projections sensitize the vestibular nuclei and modulate velocity storage function. Although selective impairment of the velocity storage mechanism has been proposed in MD, studies measuring the velocity storage function via rotational chair testing have not consistently demonstrated VSI dysfunction [40].

King et al. reported sensitization of central vestibular pathways in VM through the measurement of self-motion perception, demonstrating abnormal sensitivity to roll tilt, a stimulus that jointly engages the canal and otolith pathways and depends on velocity storage processing [41]. Head-shaking nystagmus, occurring in up to 50% of VM patients, and cross-coupled nystagmus observed during both the ictal and interictal periods may reflect amplification of peripheral vestibular asymmetry [14], as observed in several of our cases. These phenomena may result from a transient imbalance of central velocity storage related to migraine pathophysiology or peripheral vestibular deficits due to a transient neurotransmitter imbalance at the level of the vestibulocerebellum [42]. Consistent with this, rotational chair testing in patients with benign recurrent vertigo—specifically the group with HSN, 83% of whom had vestibular migraine—demonstrated that paretic HSN was associated with significantly lower VOR time constant asymmetry than reversed or absent HSN, suggesting active discharge of peripheral asymmetry through the velocity storage mechanism [43].

Additionally, VM patients report more pronounced subjective vertigo and nausea during caloric testing than individuals with other vestibular disorders, likely attributable to abnormal sensory modulation and lowered perceptual thresholds [18], potentially related to VSI dysfunction, which plays a fundamental role in motion sickness generation, a symptom more prevalent in VM than in migraine without vestibular features [44]. The convergence of peripheral vestibular insults with central migraine-related brainstem sensitization may produce caloric–vHIT dissociation through velocity storage dysfunction without overt peripheral vestibulopathy.

Limitations include the retrospective, single-center design and the higher proportion of MD relative to VM, which reflects the referral pattern of a tertiary neurotology center and may limit its generalizability. Vestibular testing was performed at a single time point during the interictal phase, precluding the assessment of the stability of the dissociation pattern over time. Delayed gadolinium-enhanced MRI, required to detect endolymphatic hydrops, and rotational chair testing, which would allow direct evaluation of velocity storage function, were not performed; consequently, the presence of hydrops and the integrity of velocity storage could not be verified. Although no patient with confirmed follow-up required reclassification, interval clinical events may have been missed in this study. Most importantly, because the seven cases were selected precisely because they exhibited caloric-vHIT dissociation, the case-level interpretations carry an inherent risk of confirmation bias and should be regarded as exploratory and hypothesis-generating. Accordingly, the contribution of this study is limited to documenting that caloric-vHIT dissociation occurs in a subset of definite VM patients and that, over long-term follow-up, these patients did not evolve toward MD—an observation supported by the data—whereas the specific mechanisms underlying the dissociation remain to be tested.

## 5. Conclusions

At the cohort level, caloric-vHIT dissociation—although characteristic of MD—was observed in approximately one in six patients with definite VM and should therefore not be regarded as a stand-alone sign of endolymphatic hydrops. At the case level, the clinical heterogeneity across our seven patients suggests that dissociation may not reflect a single process but rather several distinct mechanisms that could each give rise to a comparable pattern other than hydrops. These descriptive observations could provide a basis for future prospective studies to explore whether a distinct vestibular migraine phenotype can be defined and whether the heterogeneous mechanisms underlying this dissociation carry prognostic or therapeutic relevance. In clinical practice, interpreting this dissociation together with the auditory and longitudinal profiles, rather than in isolation, may help reduce misclassification and unnecessary MD-directed treatment.

## Figures and Tables

**Table 1:** Baseline demographic and clinical characteristics of the source cohort, by diagnostic group.

Characteristic	MD (N = 60)	VM (N = 40)
Female sex, %	55.0	87.5
Age, years (mean ± SD)	52.3 ± 12.1	48.7 ± 11.8
Disease duration, years (mean ± SD)	5.2 ± 6.0	3.4 ± 3.8
DHI score (mean ± SD)	42.8 ± 18.4	40.9 ± 27.3
VSS severity score (mean ± SD)	14.2 ± 10.4	12.4 ± 7.4
Crises in preceding year (mean ± SD)	7.2 ± 12.1	8.8 ± 12.3

MD, Ménière’s disease; VM, vestibular migraine; SD, standard deviation; DHI, Dizziness Handicap Inventory (0–100; higher = greater handicap); VSS, Vertigo Symptom Scale severity score (higher = greater symptom burden). Values reproduced from the companion cohort publication (22) and reported here descriptively to characterize the source population from which the case series was drawn.

**Table 2:** Descriptive comparison of absolute caloric sum by ear group (contextual cohort). Values are provided as descriptive effect-size estimates (Hedges’ g) to indicate the magnitude of between-group differences; no inferential hypothesis testing was performed in the present analysis, and the formal inferential comparison is reported in the companion publication.

Comparison	Hedges g
MD-affected vs. MD-non-affected	−0.56
MD-affected vs. VM	−0.80
MD-non-affected vs. VM	−0.27

MD, Ménière’s disease; VM, vestibular migraine. Negative Hedges’ g values indicate larger absolute caloric responses in VM; positive values indicate larger responses in MD.

**Table 3:** Frequency of caloric-vHIT dissociation patterns: a contextual analysis of the source cohort, with row percentages.

Diagnosis	CalHiT-0	CalHiT-A	CalHiT-B	CalHiT-C
Ménière’s disease (N = 60)	18 (30.0%)	34 (56.7%)	2 (3.3%)	6 (10.0%)
Vestibular migraine (N = 40)	32 (80.0%)	7 (17.5%)	1 (2.5%)	0 (0.0%)

Diagnosis distribution by CalHiT group (side-matched); counts with row percentages (percentage of each diagnostic group). Side-matched rule: the caloric lesser-function side is compared with the vHIT of the same side. CalHiT-0 = normal calorics and normal same-side horizontal vHIT; CalHiT-A = canal paresis (CP) ≥22% with same-side vHIT gain ≥0.80; CalHiT-B = normal calorics with same-side vHIT gain <0.80; CalHiT-C = CP ≥22% with vHIT gain <0.80. vHIT, video head impulse test.

**Table 4:** Clinical phenotype, migraine status, and longitudinal evolution in the study cohort (N = 7).

Case	Age/Sex, Migraine Phenotype	Vestibular Symptoms (Clinical)	Typical Episode Duration	Auditory Symptoms (Laterality)	BPPV (Side; Timing Relative to VM)	Context/Trigger	Long-Term Vestibular Course	Clinical Interpretation
1	69/F, low-frequency episodic migraine without aura	Recurrent spontaneous vertigo	Hours	None; no fluctuation (bilateral mild HF SNHL, non-fluctuating)	No	Remote severe vestibular event	Stable, episodic vestibular hypofunction	Compensated unilateral vestibular hypofunction with superimposed vestibular migraine
2	65/F, very-low-frequency episodic migraine without aura	Spontaneous vertigo, imbalance, falls	Minutes-hours	Tinnitus and aural fullness, bilateral, non-fluctuating	No (excluded on positional testing)	Previous head trauma	Persistent but non-progressive	Post-traumatic unilateral canal hypofunction
3	47/F, low-frequency episodic migraine without aura, catamenial	Chronic instability, spontaneous and positional vertigo	Variable	Stable tinnitus; laterality not specified in the available clinical record	Yes; right posterior and right horizontal canal; secondary, interictal, years after VM onset	Familial vestibular disorders	Stable over >10 years	Familial vestibular susceptibility without phenotypic conversion
4	50/M, low-frequency episodic migraine without aura (remote)	Episodic spontaneous and positional vertigo	Minutes	None (mild left-ear HF SNHL, non-fluctuating)	No	None identified	Complete clinical compensation	Isolated caloric weakness of uncertain clinical significance
5	18/F, very-low-frequency episodic migraine with aura	Transient spontaneous and positional vertigo	Minutes	None	No (excluded on Dix-Hallpike/supine roll test)	Pregnancy	Marked improvement	Subclinical unilateral hypofunction unmasked by migraine
6	18/F, low-frequency episodic migraine with aura	Frequent spontaneous vertigo and instability	Minutes	None	Yes; left horizontal canal; secondary, interictal, after VM diagnosis	None identified	Persistent symptoms	Multifactorial vestibular vulnerability (migraine + peripheral asymmetry + BPPV)
7	52/F, high-frequency episodic migraine with aura	Chronic head-motion dizziness, instability	Hours-24 h	Stable tinnitus, bilateral	No	None identified	Stable, low symptom burden	Compensated unilateral vestibular hypofunction with migraine modulation

F, Female; M, Male; HF, High-Frequency; SNHL, Sensorineural hearing loss; BPPV, Benign paroxysmal positional vertigo; VM, Vestibular migraine. Auditory-symptom laterality and BPPV side/timing are reported where documented in the clinical record; where laterality was not recorded, this is stated explicitly rather than inferred.

**Table 5:** Vestibular, audiological, and functional findings by case.

N	Spontaneous Nystagmus	Positional Nystagmus	HSN	Audiology (PTA)	Caloric (CP)	Caloric (DP)	vHIT	VEMPs (cerv/oc)	Other/CalHiT
1	–	–	–	R/L: 22/22.5 HF dB	RCP 2%	LDP 8%	↓ gain 0.62 (L; R 0.97)	Normal/normal	–/B
2	Horizontal	–	Horizontal	Normal	RCP 81%	LDP 5%	Normal (R 0.97; L 0.82)	Normal/normal	–/A
3	Horizontal	+	–	Normal	RCP 54%	LDP 8%	Normal (R 1.05; L 0.99)	Normal/Oc↓62% (R)	BPPV/A
4	DBN	–	DBN	R/L: 8/22.5 HF dB	LCP 42%	RDP 22%	Normal (R 0.92; L 0.89)	Normal/normal	–/A
5	DBN	–	Horizontal	Normal	LCP 63%	LDP 17%	Normal (R 0.93; L 0.80)	Normal/normal	–/A
6	–	+	–	Normal	RCP 49%	RDP 2%	(R 0.93; L 0.88)	Normal/normal	BPPV/A
7	–	+	–	Normal	LCP 90%	LDP 30%	Normal (R 0.93; L 0.87)	Normal/normal	–/A

R, right; L, left; SNHL, Sensorineural hearing loss; BPPV, Benign paroxysmal positional vertigo; LCP, Left canal paresis; RCP, Right canal paresis; LDP, Left directional preponderance; RDP, Right directional preponderance; DBN, Downbeat nystagmus; HSN, Head-shaking nystagmus; HF, High frequency; PTA, Pure-tone audiometry (conventional average at 0.5, 1, 2 and 3 kHz; HF SNHL refers to elevated thresholds at 4–8 kHz); vHIT, Video head impulse test; CP, Canal paresis; DP, Directional preponderance; VEMPs, Vestibular evoked myogenic potentials (cervical/ocular); CalHiT, caloric-vHIT pattern. Downward arrows (↓) denote a reduced response below the normal reference threshold (horizontal vHIT gain <0.80; VEMP amplitude reduction relative to the contralateral side). Case 4 vHIT values are reported here and in the corresponding case description in Section 3.2.

## Data Availability

The data supporting the findings of this study are contained within the article. Further inquiries can be directed to the corresponding author.
